# Editorial: Insights in emotion science

**DOI:** 10.3389/fpsyg.2025.1643052

**Published:** 2025-07-17

**Authors:** Florin Dolcos, Andrew Kemp, Alfons O. Hamm

**Affiliations:** ^1^Beckman Institute, Psychology Department, and Neuroscience Program, University of Illinois at Urbana-Champaign, Urbana, IL, United States; ^2^School of Psychology, Faculty of Medicine, Health and Life Science, Swansea University, Swansea, United Kingdom; ^3^Institute of Psychology, University of Greifswald, Greifswald, Germany

**Keywords:** emotional memory, emotional distraction, affective neuroscience, emotion-cognition interaction, affective sciences, affective disorders, social cognition, psychology advances

This century has witnessed unprecedented increasing interest and growth in the field of *Emotion Science*. The goal of this special edition Research Topic (RT) was to (1) shed light on recent progress made in this field, by providing a thorough overview of the state of the art in this area of research, (2) identify the greatest challenges in its various sub-disciplines, and (3) propose solutions addressing these challenges. This RT solicited forward-looking contributions from the editorial board members that would inspire, inform, and provide direction and guidance to researchers in the field. A total of 12 manuscripts have been accepted for inclusion in this RT, covering a wide variety of topics—from aspects related to emotion-cognition interactions in healthy functioning and clinical conditions to the role of Artificial Intelligence in emotion processing—and manuscript formats (reviews, empirical reports, opinion, articles, etc.).

The 12 contributions can be organized around two main loosely defined themes: *Emotion-Cognition-Behavior Interactions* (7 articles) and *Emotion Processing in Social Contexts* (5 articles). Regarding the first theme, central to the efforts in the field are investigations of emotion-cognition interactions and the associated neural mechanisms (Figure 1). Five articles of the present RT focus more specifically on aspects circumscribed by this general area of research. First, the review by Dolcos et al. discusses opposing effects of emotion on cognition at multiple levels of analysis and emphasizes the need to consider the various factors that can influence enhancing and impairing effects of emotion on cognition. Although identification of a coherent theoretical framework that covers all levels of emotion-cognition interactions was beyond its scope, the review by Dolcos et al. pointed to emerging theoretical accounts resulted from research aimed at reconciling divergent patterns in specific domains. For instance, it introduces the readers to a new model of dual enhancement of associative memory by emotion (i.e., the *DEAME* model; Bogdan et al., 2024).

**Figure 1:**
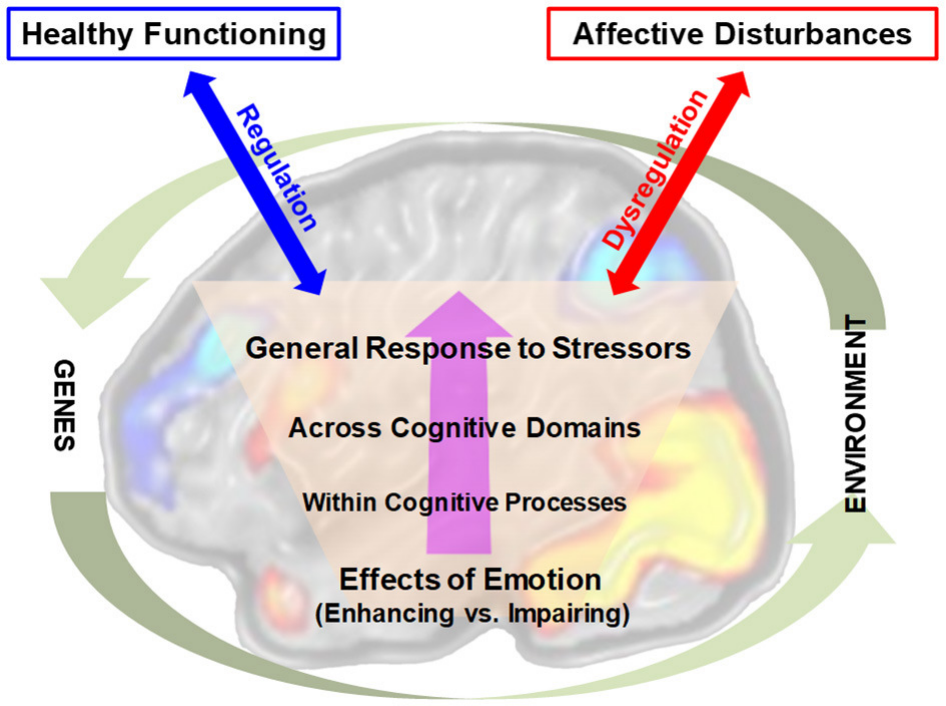
Emotion-cognition interactions in the brain and their relation to adaptive and maladaptive outcomes. The diagram illustrates opposing effects of emotion on cognition at increasing levels of complexity in emotion-cognition interactions. The involvement of brain mechanisms at all these levels is suggested by the background brain image. The effective vs. dysfunctional engagement of regulatory mechanisms in emotion-cognition interactions are depicted by the blue and red arrows, linked to adaptive vs. maladaptive outcomes, respectively. Finally, these interactions occur in the larger context circumscribed by interplays between genetic and environmental factors influencing them. Adapted from Dolcos et al., with permission.

Second, also investigating emotion-memory interactions, the mega-analysis by Ventura-Bort et al. specifically focusses on the impact of emotion on source memory, based on data from multiple studies using a similar design. The results point to dissociating effects of arousal on memory for contextual information of events (source memory) vs. memory for specific neutral aspects of events (item memory). Namely, consistent with available evidence (reviewed in Bogdan et al., 2024), results showed a recollection-based retrieval advantage for pleasant and unpleasant source contextual details, but there was no evidence for significant influence of the context's affective category on item memory. Third, on a related topic regarding the impact of emotion on cognition, but in the context of distracting effects (Iordan et al., 2013), the study by Ferrari et al. investigated whether the behavioral interference of emotional distraction is attenuated following repeated (trained) exposure to novel task-irrelevant emotional pictures. Results showed attenuated interference from emotional distracters after sustained training, but the electrophysiological markers of affective processing, as measured with EEG recordings, were unaffected by the training. This points to a possible dissociation between behavioral and neural effects in the impact of emotional distraction.

Two other articles expand the discussion of emotion-cognition-behavior interactions specifically linked to the impact of emotion on decision-making. The perspective article by Buelow et al. examines decision-making as a complex executive function involving affective, cognitive, and personality aspects, and also proposes that reconceptualization of decision-making by integrating affective and cognitive aspects can improve task psychometrics and clinical utility. The authors identify limitations of previous decision-making research (low validity and reliability of the tasks) and also provide suggestions for improvements, including the need to: (a) assess decision-making as a specific cognitive ability, (b) further assess the test–retest reliability of decision-making tasks, and (c) reimagine future research by considering implications for both basic research and clinical investigations. The mini review by Ha and Lim explores the link between emotion and eating decisions and behaviors. The authors discuss emotional eating as linked to disinhibited decisions driven by heightened reward values to eat palatable foods, in response to negative emotions and social isolation. Emotional eating is also examined as a potentially maladaptive coping strategy under negative emotion and stress, linked to dysfunctional interactions between the brain reward system (involved in hedonic eating decisions) and the brain systems associated with executive control (involved in health-oriented eating decisions).

The last two contributions of this theme explore basic physiological and behavioral effects of emotion associated with two fundamental dimensions of affective stimuli and experiences: *arousal* (i.e., intensity) and *valence* (i.e., pleasantness/unpleasantness; Russell, 1980). Focusing on physiological responses associated with experienced affective states, the study by Leung and Romano challenges the common notion that the Autonomous Sensory Meridian Response (ASMR), associated with goosebump-inducing situations, is exclusively interpreted as linked to positive valence. Instead, their findings show that ASMR can occur linked to both positive and negative emotional situations, suggesting a more general link to emotional arousal rather than to positive valence only. The authors also highlight the therapeutic relevance of considering valence-related differences in experiencing and interpreting ASMRs. The article by Marin and Gingras discusses, through evolutionary lenses, how music-induced emotions can influence sexual attraction and behavior, and highlights how vocalizations and music contribute to the communication of emotions. The authors also point to the music's ability to increase emotional arousal and influence mate selection and aesthetic display, and also discuss evidence of cross-modal transfer of arousal from music to other sensory/perceptual domains. This report also suggests investigation of this topic through triadic interactions of emotion (through music), cognition, and decision-making.

Regarding the second main theme covered in the present RT, three of the contributions cover more generic aspects of social cognition and learning in healthy functioning and clinical groups and the other two specifically focus on empathy. First, the article by Jellema et al. emphasizes social intuition as instrumental in successful human interactions, with a focus on the implicit, involuntary, nature of social intuition, rather than on higher-level explicit Theory-of-Mind processes. The authors argue that traditional implicit learning tasks are insufficient due to their lack of social context and affective components, and propose a new paradigm associating valences with identities through implicitly learned bodily cues. This article also discusses neural mechanisms associated with social intuition (i.e., the mirror neuron system) and also points to clinical conditions in which impairments in implicit social/affective learning are relevant (e.g., autism spectrum disorder). Second, the study by Namba et al. used computational models to investigate value learning and detection of emotionally-valued neutral faces in young and older adults. The authors conclude that the sensitivity of learning feedback decreases with age. They found that the learning rates for reward and punishment were higher for younger than for older participants, who also showed reduced sensitivity to negative faces. Namely, older participants did not show different learning rates between reward and punishment trials, unlike the young participants who had higher learning rate parameter values for punishment than for reward trials. Third, the research by Abo Foul et al. examined emotion perception from incongruent face-body composites in three groups: Parkinson's (PD), schizophrenia (SZ), and healthy controls (HC). When presented with conflicting cues, the PD group tended to categorize the emotion based on the body expression, whereas the HC group relied more on the facial expression and the SZ group showed no consistent prioritization pattern. These findings were independent of the ability to recognize isolated face or body emotions, cognitive function, depression, or motor symptoms in the PD and SZ groups, and have implications for the way these individuals interpret emotions in others.

Finally, the last two contributions of this theme focus on empathy in humans and AI agents. The review by Mansur and DeFelipe explores empathy from an artistic perspective, as a fundamental way of conveying emotions in humans. Basing their discussion of empathy on da Vinci's work (both in arts and human anatomy), the authors also make connections to the German romanticism, renaissance, and the philosophy of art creation. The authors also highlight the importance of interdisciplinary approaches in social neuroscience, to explore the neural basis of empathy, thus bringing us closer to realizing da Vincis's vision of uniting artistic perception with scientific explanation. On the other hand, the opinion article by Tagesson and Stenseke discusses the skepticism surrounding the empathic abilities of AI agents, particularly related to the negative perception arising when people realize that the empathy is AI-generated. Inspired by the correspondence article by Perry (2023), and consistent with a broader aversion toward the prospect of artificial empathy (AE), the authors suggest that human attitudes toward AI can change to perceive AE as genuine. They argue that, as AI sophistication increases and human attitudes evolve, [aspects of] AE could be seen as “real empathy.”

Overall, we are confident that this eclectic collection of contributions from editorial board members of *Emotion Science* will inform, inspire, and provide direction and guidance to researchers in the field.

## References

[B1] Bogdan P. C. Dolcos F. Katsumi Y. O'Brien M. Iordan A. D. Iwinski S.. (2024). Reconciling opposing effects of emotion on relational memory: Behavioral, eye-tracking, and brain imaging investigations. J. Exp. Psychol. Gen. 153, 3074–3106. 10.1037/xge0001625 39298200

[B2] Iordan A. D. Dolcos S. Dolcos F. (2013). Neural signatures of the response to emotional distraction: a review of evidence from brain imaging investigations. Front. Hum. Neurosci. 7:200. 10.3389/fnhum.2013.00200 23761741 PMC3672684

[B3] Perry A. (2023). AI will never convey the essence of human empathy. Nat. Hum. Behav. 7, 1808–1809. 10.1038/s41562-023-01675-w 37474839

[B4] Russell J. A. (1980). A circumplex model of affect. J. Pers. Soc. Psychol. 39, 1161–1178.

